# Exploring the Extraction Methods of Phenolic Compounds in Daylily (*Hemerocallis citrina* Baroni) and Its Antioxidant Activity

**DOI:** 10.3390/molecules27092964

**Published:** 2022-05-05

**Authors:** Zhilin Hao, Li Liang, He Liu, Yi Yan, Yuyu Zhang

**Affiliations:** Beijing Key Laboratory of Flavor Chemistry, School of Light Industry, Beijing Technology and Business University (BTBU), Beijing 100048, China; hzl15716324037@163.com (Z.H.); gcfll@126.com (L.L.); liuhe6660@163.com (H.L.); zhangyuyu@btbu.edu.cn (Y.Z.)

**Keywords:** daylily, phenolic compounds, antioxidant, LC-QTOF-MS/MS

## Abstract

Daylily is a valuable plant resource with various health benefits. Its main bioactive components are phenolic compounds. In this work, four extraction methods, ultrasonic-assisted water extraction (UW), ultrasonic-assisted ethanol extraction (UE), enzymatic-assisted water extraction (EW), and enzymatic-assisted ethanol extraction (EE), were applied to extract phenolic compounds from daylily. Among the four extracts, the UE extract exhibited the highest total phenolic content (130.05 mg/100 g DW) and the best antioxidant activity. For the UE extract, the DPPH value was 7.75 mg Trolox/g DW, the FRAP value was 14.54 mg Trolox/g DW, and the ABTS value was 15.37 mg Trolox/g DW. A total of 26 phenolic compounds were identified from the four extracts, and the UE extract exhibited a higher abundance range of phenolic compounds than the other three extracts. After multivariate statistical analysis, six differential compounds were selected and quantified, and the UE extract exhibited the highest contents of all six differential compounds. The results provided theoretical support for the extraction of phenolic compounds from daylily and the application of daylily as a functional food.

## 1. Introduction

Phenolic compounds, as secondary plant metabolites, are widely present in fruits and vegetables. They have antioxidant, anti-inflammatory, and various other biological effects that can prevent a number of diseases including cardiovascular disease and cancer [1]. Phenolic compounds generally contain two main categories of components: flavonoids and non-flavonoid polyphenols [2]. Flavonoids can be further divided into flavones, flavonols, flavononse, flavanols, and isoflavones. Phenolic acids are a type of non-flavonoid polyphenol, generally classified as hydroxybenzoic acids and hydroxycinnamic acids [3]. As a result of the potential adverse health effects of synthetic antioxidants, natural antioxidants are gradually replacing synthetic antioxidants [4]. Daylily (*Hemerocallis citrina* Baroni), which is widely distributed in Southeast Asia, has long been used as a medicine and a favored vegetable [5]. Daylily exhibits strong antioxidant activities both in vitro and in vivo [6]. This beneficial effect can be attributed, at least partially, to the action of antioxidant compounds such as phenolic compounds [7]. Gao et al. (2017) compared the contents of free phenolic acids and conjugated phenolic acids in seven different vegetables, and the results showed that daylily had the highest contents of free phenolic acids and conjugated phenolic acids [8].

Extraction is an essential step in the study of natural antioxidants. The choice of extraction method has an important impact on the extraction of phenolic compounds. Mao et al. (2006) used water and ethanol to extract the phenolic compounds from daylily with different drying methods [9]. They demonstrated that no matter what kind of drying methods were adopted, ethanol daylily extracts possessed higher antioxidant activity and phenolic content than those of water extracts. Ultrasonic-assisted extraction (UAE) is considered to be an effective extraction method in the food and pharmaceutical industries and has been applied to extract flavonoids from daylily [10,11], with a total of 22 flavonoids in the extracts being identified through HPLC-ESI-TOF-MS. Enzymatic-assisted extraction (EAE) enhances the release of bioactive compounds due to the high specificity and efficiency of its enzymes. Cellulases and pectinases can degrade and disrupt the structural integrity of plant cell walls. Zhang et al. [12] extracted phenolic compounds from walnuts using EAE and based on the results of LC-MS and principal component analysis (PCA), a total of 32 phenolic compounds were identified, with the most abundant being ellagic acid.

Although the daylily has been deemed a good source of phenolic compounds in recent works, none of these has ever reported the effects of different extraction methods on the phenolic profile of the obtained daylily extracts. Therefore, the present study aimed to assess the antioxidant activities and the related phenolic compounds in daylily via different extraction methods. In this work, phenolic compounds were extracted from daylily using four methods: ultrasound-assisted water extraction (UW), ultrasound-assisted ethanol extraction (UE), enzyme-assisted water extraction (EW), and enzyme-assisted ethanol extraction (EE). The phenolic compounds in the four extracts were analyzed using liquid chromatography tandem quadrupole time-of-flight mass spectrometry (LC-QTOF-MS/MS). Furthermore, the antioxidant activities of the daylily extracts prepared using different extraction methods were further evaluated. These results are useful for the potential application of daylily as a functional food.

## 2. Results and Discussion

### 2.1. The Effect of Extraction Method on TPC

**Figure 1:**
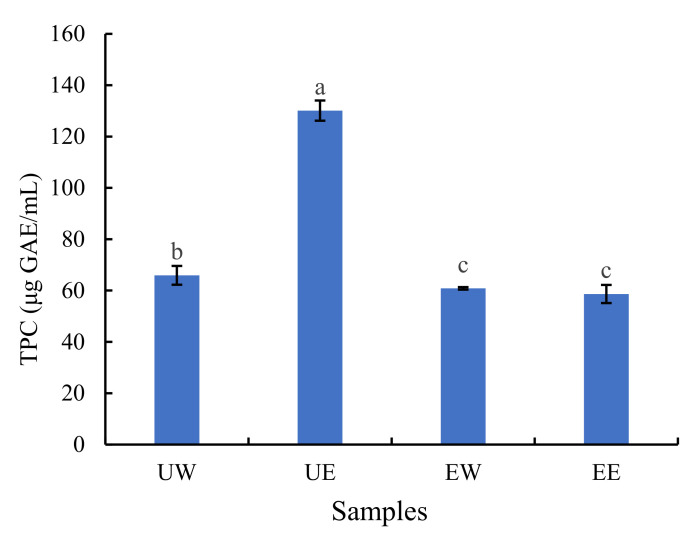
The total phenolic content (TPC) among the four different extracts. Different letters represent significant differences according to Duncan’s test (*p* < 0.05). UW = ultrasound-assisted water extraction, UE = ultrasound-assisted ethanol extraction, EW = enzymatic-assisted water extraction, EE = enzymatic-assisted ethanol extraction.

The total phenolic content (TPC) of the four extracts was calculated and shown in Figure 1. The yields from the UW, UE, EW, and EE extracts are shown in Table S1 from Supplementary Materials. Although the EE yield (44.96%) was the highest among the four extracts, the UE extract had the highest TPC content at 130.05 mg/100 g DW (dry weight). The TPC of the UE extract was significantly (*p* < 0.05) higher than that of the EE extract, and a significantly (*p* < 0.05) higher TPC for the UW extract was observed when compared to the EW extract. On the basis of the TPC results, UAE was more efficient than EAE in water and 50% ethanol solvent, which was consistent with the TPC value of acerola fruit [13]. At the lab scale and pilot-plant scale, UAE has been shown to extract natural products with better yields than conventional techniques [14]. During UAE, the cavitation process can cause cell swelling or cell wall rupture, which is conducive to the release of phenolic compounds [15]. Phenolic compounds have the characteristics of low vapor pressure and Henry’s coefficient. These properties prevent the diffusion of phenol molecules into the cavitation bubble, so it remains in the bulk of the solution during the cavitation process [16]. For EAE, previous studies have shown that temperature affects the stability and biological activity of flavonoids. According to their structures, flavonoids are more or less sensitive to heat treatment [17]. During enzyme inactivation, direct heating at 95 °C inevitably affects flavonoids, and thereby affects the TPC. According to the literature, the *Hemerocallis* plants have high contents of phenolic acid and flavonoids [18]. However, high-temperature heat treatment may cause the loss of phenolic compounds in daylily. This may be the reason why UAE was better than EAE at the same treatment time. The TPC of the UE extract was significantly (*p* < 0.05) higher than that of the UW extract, which is consistent with the results of previous research on the extraction of polyphenols with different solvents [19,20]. Solvent polarity plays a key role in increasing the solubility of phenolic components [21]; the preference of the phenolic compounds for ethanol may be caused by their non-polar part and the aliphatic fragment of alcohols [22,23]. This is also similar to previous studies demonstrating that a combination of water and organic solvents may help improve the efficiency of extraction [24]. However, there was no significant difference in the TPC between the EW extract and the EE extract, which may be related to the degradation of flavonoids caused by the high-temperature treatment.

### 2.2. The Effect of Extraction Method on Antioxidant Activity

To evaluate antioxidant efficiency, different methods specific to their chemical properties were applied. In this work, three complementary methods were chosen to evaluate the antioxidant activity due to their simplicity, stability, and accuracy.

**Figure 2:**
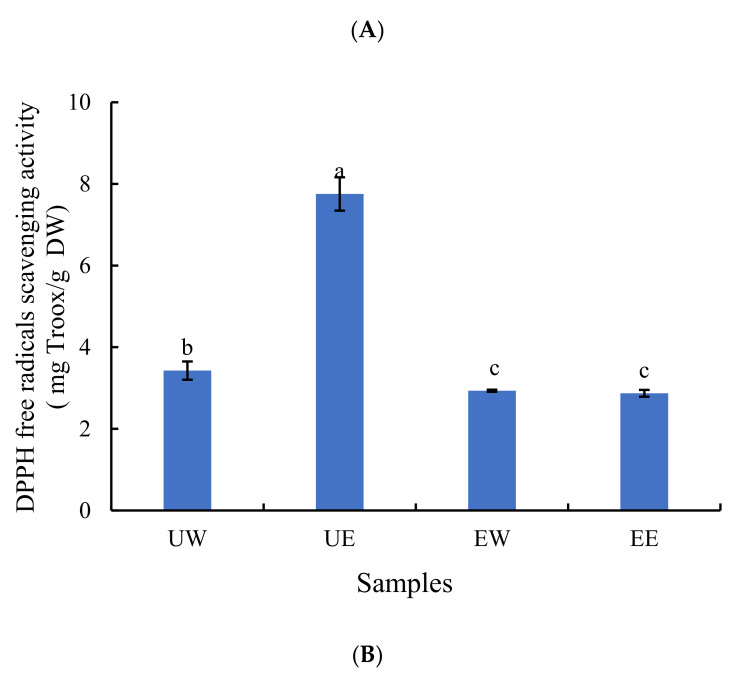

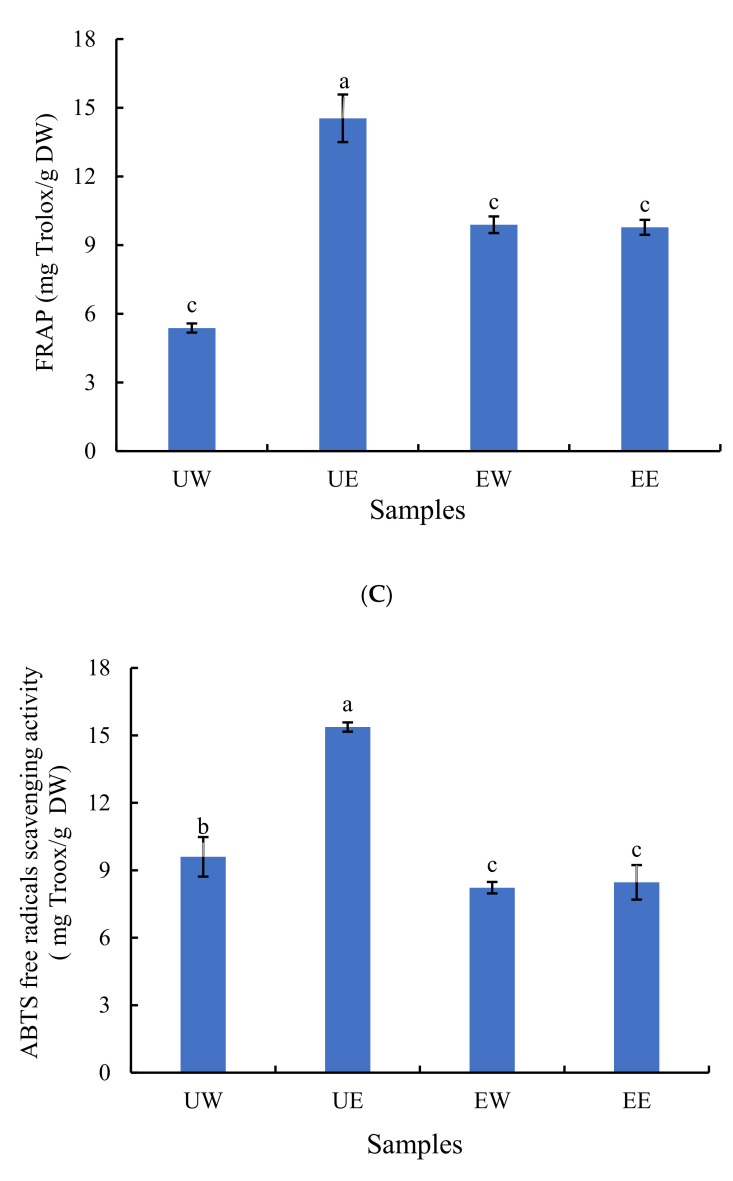
The determination of the three antioxidant indices in the four different extracts. (**A**) DPPH free radicals scavenging activity, (**B**) FRAP value, and (**C**) ABTS free radicals scavenging activity. Different letters represent significant differences according to Duncan’s test (*p* < 0.05). UW = ultrasound-assisted water extract, UE = ultrasound-assisted ethanol extract, EW = enzymatic-assisted water extract, EE = enzymatic-assisted ethanol extract.

The DPPH free radical scavenging activities of the four daylily extracts are shown in Figure 2A. The antioxidant activity of the UE extract in scavenging DPPH was 7.75 mg Trolox/g DW, which was significantly (*p* < 0.05) higher than that of the other three groups, and the scavenging DPPH of the UW extract was significantly higher (*p* < 0.05) than that of the EW extract and EE extract. The scavenging DPPH was positively correlated with the TPC. A linear correlation between scavenging DPPH and the concentrations of phenolic compounds in various vegetables has been reported [25]. It is well established that the free radical scavenging activity of plant extracts mainly depends on phenolic compounds. In this experimental group, UAE accelerated the dissolution of phenolic compounds, resulting in a higher DPPH value in the extracts. The number of hydroxyl groups available in the reaction medium increased. Therefore, the possibility of hydrogen donation to free radicals was enhanced by increasing the concentrations of phenolic compounds [26]. 

The FRAP assay provides a simple and effective method for measuring the ability of antioxidants to act as reducing agents in plant samples [26]. Figure 2B shows that the UE extract exhibited the highest FRAP value (14.54 mg Trolox/g DW), which was significantly (*p* < 0.05) higher than those in the other three groups because of its higher TPC. In addition, the FRAP values of the EW (9.89 mg Trolox/g DW) and EE extracts (9.78 mg Trolox/g DW) were substantially (*p* < 0.05) higher than that of the UW extract. Although the TPC in the UW extract was higher than that of the EW or EE extract, the enzymatic hydrolysis process was able to promote the release of other antioxidant substances, thereby strengthening the antioxidant capacity of the EW and EE extracts [27].

The ABTS assay is another widely used method to determine the anti-radical scavenging ability of phenolic compounds based on their hydrogen atom contribution trend. Figure 2C indicates that the UE extract had the highest ABTS free radical scavenging activity (15.37 mg Trolox/g DW) among the four extracts. Moreover, the ABTS free radical scavenging rate of the UW extract was significantly higher (*p* < 0.05) than that of the EW or EE extract. This is consistent with the results of the DPPH free radical scavenging ability and the TPC, but inconsistent with what is implied by the FRAP value.

It is well-known that phenolic compounds are effective free radical scavengers and antioxidants. As a result, attempts were made to analyze the correlation between the concentration of phenolic compounds and the antioxidant activities using Pearson’s correlation coefficient (R^2^). The TPC in the four extracts exhibited a strong correlation with the DPPH, ABTS, and FRAP activities (R^2^ = 0.998, 0.986, and 0.773, respectively, *p* < 0.05). This result is consistent with previous research regarding the correlation between ABTS, DPPH, and FRAP in 133 medicinal plants [28]. There may be a certain relationship in terms of the principles of the three different measurement methods [29]. Moreover, the FRAP value is more susceptible to interference from reaction kinetics and quantitative methods than ABTS and DPPH. 

### 2.3. Identification of Phenolic Compounds

**Figure 3:**
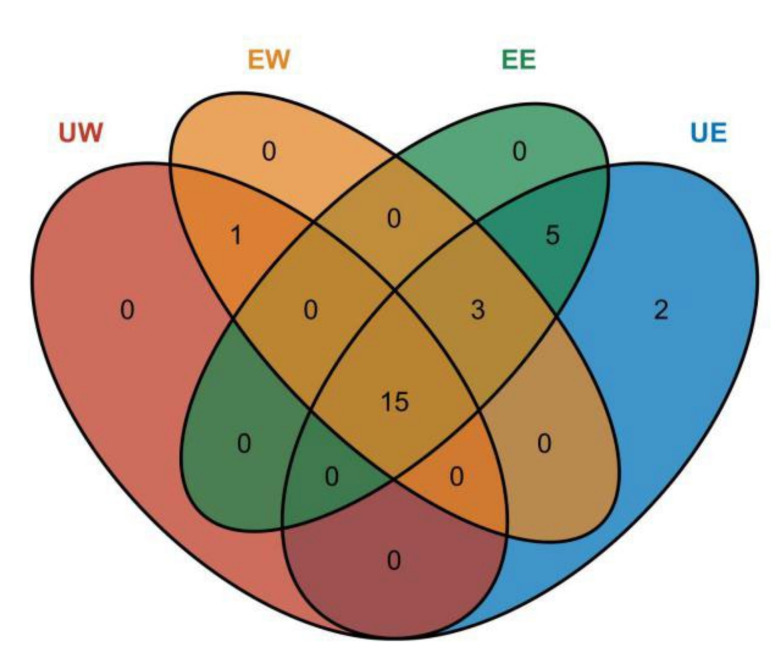
Venn diagram of phenolic compounds identified in the four extracts.

The main product ions in negative mode (*m/z*) and the molecules proposed corresponding to each chromatographic retention time from the four extracts are listed in Table 1. The chemical structures of twenty-six phenolic compounds and their fragmented forms at this ion source energy are presented in Figure S1 from Supplementary Materials. According to the ion fragmentation information of the compound, twenty-six types of phenolic compounds were identified in the four extracts including eight phenolic acids and eighteen flavonoids. The phenolic compounds detected in UE, EE, EW, and UW extracts were 25, 23, 19, and 16, respectively. For daylily samples, 15 phenolic compounds were detected in the four extracts. The uniqueness and overlapping characteristics of the compounds using different methods are shown in Figure 3. Types of phenolic compounds identified using LC-QTOF-MS/MS are presented in Table 1. In a previous study, chlorogenic acid, rutin, and quercetin were found in daylily samples [9]. These three phenolic compounds were also detected in this experimental sample. Szewczyk et al. [18] analyzed and identified the phenolic compounds in the *Hemerocallis* plant using UPLC-ESI-MS. The types of phenolic compounds identified in the work were consistent with those identified in this experiment including kaempferol, rutin, etc. Obvious differences in the types and contents of compounds extracted using different methods were also observed. Artemisinin was only detected in the samples from the UW extract and EW extract, while the quercetin, hesperetin, vanillin, caffeic acid, and 3,4-bihydroxybenzoic acid were detected in the UE and EE extracts. Naringenin, phytoside, and chlorogenic acid were not detected in the UW extract alone; however, isorhamnetin and kaempferol were only detected in the UE extract. This is further supported by previous studies, which showed that the extraction methods had an impact on phenolic compounds [12] and it was easier to extract the majority of phenolic compounds from plants using the ethanol extraction technique.

### 2.4. Analysis of Differential Compounds

**Figure 4:**
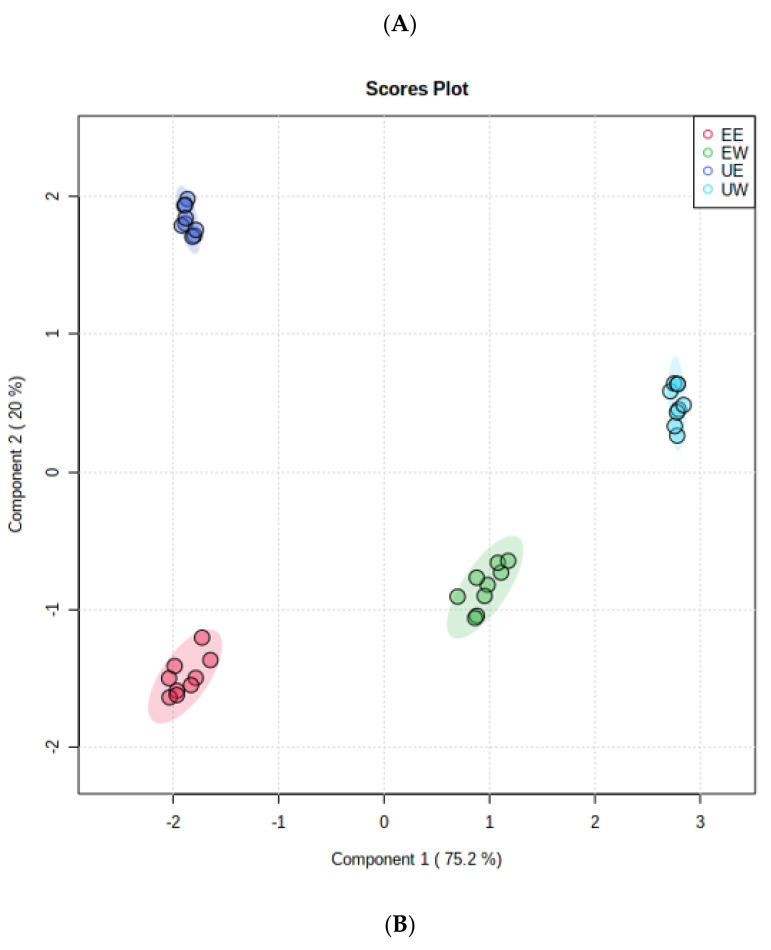

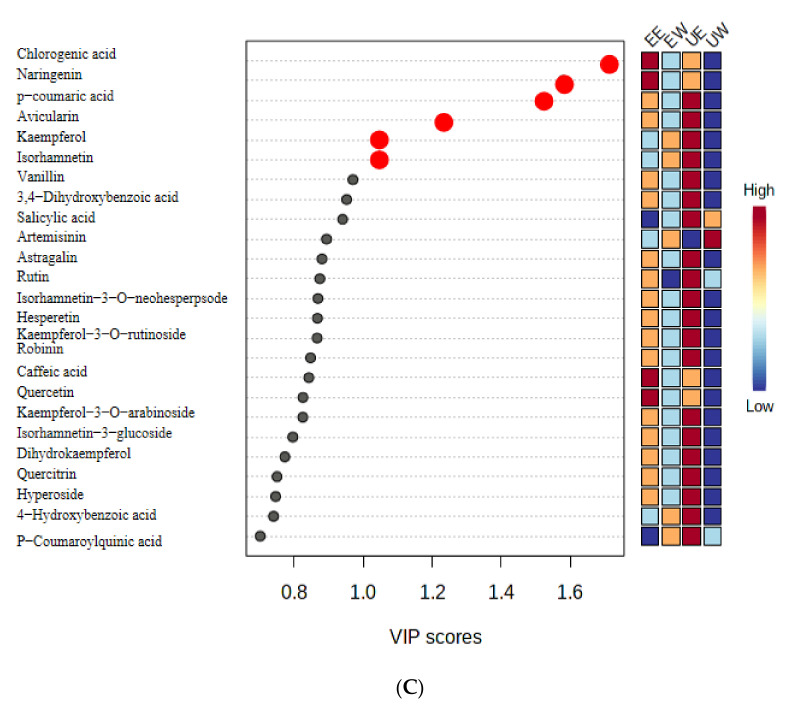

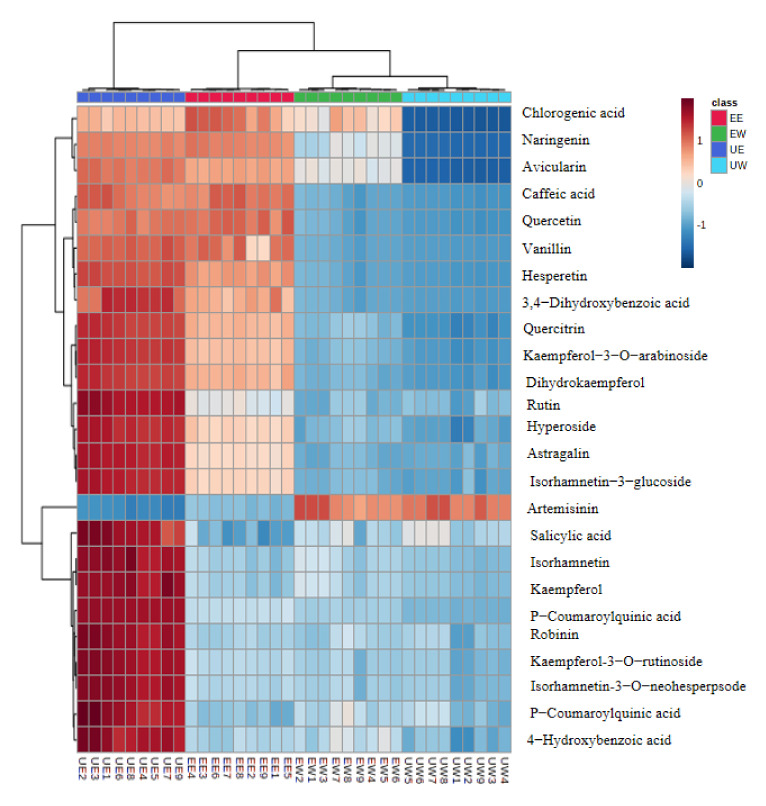
PLS-DA, VIP scores, and heat map of the four extracts in negative mode. (**A**) Scores plot of PLS-DA, (**B**) VIP scores, and (**C**) Heat map.

Multivariate statistical analysis was used to identify differential compounds between the samples [30]. In the UW, UE, EW, and EE extracts, a total of 26 phenolic compounds were identified by comparing the MS/MS spectra from the MassBank database (https://massbank.eu/MassBank/ accessed on 20 June 2021) and databases built from standard compounds. A partial least squares discrimination analysis (PLS-DA) was further applied in the data analysis. The PLS-DA score plots are presented in Figure 4A. The total variance in the data represented by the first two principal components was 95.2%. A clear distribution trend among the four groups can be observed. Differential compounds were then selected according to the parameter VIP > 1 and *p* value < 0.05 from PLS-DA. As shown in Figure 4B, six phenolic compounds (marked in red) were selected as the differential compounds between the four groups including four flavonoids (naringenin, avicularin, kaempferol, isorhamnetin) and two phenolic acids (chlorogenic acid, p-coumaric acid). Furthermore, the six differential compounds were quantified, and the results are presented in Table 2. In the UE extract, the contents of the six differential compounds were significantly higher than those in the other extracts. Chlorogenic acid (CAG) had the highest VIP score and was quantified in UE (6.713 ± 0.097 mg/100 g DW), EW (6.170 ± 0.153 mg/100 g DW), and EE (6.406 ± 0.174 mg/100 g DW) extracts. As one of the most available and better-known phenolic acid compounds in the human diet, CAG has anti-inflammatory, antioxidant, and hepatoprotective effects [31,32,33]. In different systems, the anionic form of CAG achieves its antioxidant activity through hydrogen atom transfer-radical adduct formation, sequential proton loss electron transfer, and single electron transfer-proton transfer [34,35]. Naringenin (4,5,7-trihydroxy flavanone) was a common flavonoid aglycone of naringin [36]. The antioxidant activity of naringenin results from its molecular structure (i.e., it contains three hydroxyl substituents), which are highly reactive to reactive oxygen species and reactive nitrogen [37]. The naringenin content in the UE extract was the highest (2.759 ± 0.075 mg/100 g DW), and the absence of naringenin may be attributed to its poor water solubility. Trans-4-hydroxycinnamic acid, also called p-coumaric acid, is a polyphenol precursor, especially for the formation of flavonoids, flavones, and flavonols [38]. The p-coumaric acid content is stable at room temperature but decreases when the temperature reaches 75 °C [39]. In this experiment, the p-coumaric acid content in the UE extract (1.091 ± 0.012 mg/100 g DW) was higher than that of the EE extract (0.952 ± 0 mg/100 g DW). In addition, kaempferol and isorhamnetin were only detected and quantified in the UE extract, and the contents were 9.592 ± 0.167 mg/100 g DW and 2.126 ± 0.015 mg/100 g DW, respectively. Among the six differential compounds, kaempferol content was the highest in the UE extract, which may contribute to its favorable antioxidant activity.

A heat map was utilized to directly show the trends of phenolic compounds identified in the daylily extracts. As shown in Figure 4C, except artemisinin, the content of phenolic compounds in the UE extract was higher than those of the other three extracts, which was also consistent with the determination of the TPC. Among them, chlorogenic acid, naringenin, and hyperoside had the lowest content in the UW extract. Through the heat map analysis, the four different extraction methods of daylily samples could be distinguished, and the UE extracts were significantly different from the other three groups. The six differential compounds exhibited the highest trend in terms of compound content in the UE extract on the heat map, followed by the EE extract. The different distribution of phenolic compounds in the four extracts can be attributed to their different structural characteristics and properties.

## 3. Materials and Methods

### 3.1. Materials and Reagents

Dried daylily was purchased from Yonghui Market (Beijing, China). Methanol and acetonitrile (hypergrade) were purchased from Merck (Darmstadt, Germany). Ethanol was purchased from Sinopharm Chemical Reagent Co. Ltd. (Shanghai, China). Cellulase and pectinase with the enzyme activity of 100,000 U/g were purchased from Pangbo Biological Co. Ltd. (Nanning, China). Ultra-pure water was purchased from Watsons Group Co. Ltd. (Hongkong, China). The kit (G0117F) was purchased from Suzhou Grace Biotechnology Co. Ltd. (Suzhou, China). 

### 3.2. Extraction Methods of Daylily

The daylily was pulverized using a pulverizer (BJ-400T, Huzhou, China) to create sample particles with a particle size of 80 mesh.

#### 3.2.1. Ultrasonic-Assisted Extraction (UAE)

Ultrasonic-assisted ultra-pure extraction was applied to extract phenolic compounds from daylily powder. The processing conditions were as follows: sixty grams of sample was mixed with 300 g of ultra-pure water. The sample was placed in brown bottles with narrow necks and immersed in an ultrasonic water bath for 120 min at the temperature of 30–35 °C (KQ-400DE, Kunshan, China) at 400 W. Then, the obtained extracts were centrifuged at 10,000 r/min for 5 min at 4 °C, and the supernatant was collected and lyophilized to obtain the extract (UW). The powder sample was stored at 4 °C for further research. The daylily powder (UE) extract was obtained by ultrasonic-assisted ethanol extraction. The treatment conditions were the same as those described above for ultrasonic-assisted water extraction, but the solvent was changed to 50% ethanol.

#### 3.2.2. Enzymatic-Assisted Extraction (EAE)

According to the literature, cellulase and pectinase are often used in the extraction of plant phenolic compounds [6]. The enzymatic extraction conditions were as follows: sixty grams of sample was mixed with 300 g of ultra-pure water. The enzymatic hydrolysis required a constant reaction temperature of 50 °C. When the sample reached 50 °C, 0.03 g of cellulase and 0.03 g of pectinase were added, and the sample was enzymatically hydrolyzed for 2 h. Then, enzymes were inactivated at 95 °C for 15 min. Furthermore, the obtained extract was cooled, centrifuged at 10,000 r/min for 5 min at 4 °C, and the supernatant was collected and lyophilized to obtain the extract (EW). Enzymatic-assisted ethanol extraction was applied to obtain the extract (EE) from daylily. The processing conditions were the same as the above-mentioned enzymatic water extraction process, but after adding 50% ethanol solution, the enzymatic hydrolysis solution was completely removed.

### 3.3. Determination of Total Phenolic Content (TPC)

The TPC of samples was measured with a kit (G0117F). This kit included reagent 1 (modified Folin–Ciocalteu reagent) and reagent 2 (Na_2_CO_3_). 40 μL (0 μL for the blank group) of the extract solution (5 mg/mL) were mixed with reagent 1 (2 mL), and the mixtures were allowed to stand for 30 min in the dark at room temperature. Then, 1 mL of reagent 2 was added to each sample and 360 μL H_2_O (400 μL H_2_O for the blank group) was supplemented in each sample. The absorbance was measured at 760 nm.

### 3.4. Determination of Antioxidant Indexes

A total of 0.1 g of the sample was dissolved in 10 mL of water to obtain 0.01 g/mL of the sample solution and the antioxidant capacities were then determined.

#### 3.4.1. Ferric Reducing/Antioxidant Power Assay (FRAP Assay)

The FRAP method is often used to test the total antioxidant capacity of plants, Chinese herbal medicine extracts, and various antioxidant solutions. In an acidic environment, antioxidants can reduce Fe^3+^-TripyridineTriazine (Fe^3+^-TPTZ) to produce the blue substance Fe^2+^-TPTZ. The FRAP was determined according to the method described previously [40]. The absorbance was measured at 593 nm. Trolox was used as the standard, and the FRAP results were expressed as mg Trolox equivalents per gram of DW (mg Trolox/g DW).

#### 3.4.2. DPPH Free Radical-Scavenging Assay

DPPH radical has a strong absorption at 517 nm, and its methanol solution is purple. With the reaction of the antioxidant substance, its color becomes lighter, and then the DPPH scavenging ability in the sample can be quantitatively analyzed. The DPPH radical scavenging activity was determined according to the previous method with some modifications [18]. Absorbance was measured at 517 nm. Trolox was used as the standard, and the DPPH free radical scavenging assay results were expressed as mg Trolox equivalents per gram of DW (mg Trolox/g DW).

#### 3.4.3. ABTS Free Radical Scavenging Ability

ABTS radical scavenging activity was determined using the ABTS assay according to the method of Binsan et al. (2008) [41] with slight modification. A pure ethanol sample was the control group. Moreover, absorbance was measured at 734 nm. The ABTS free radical scavenging ability results were expressed as mg Trolox equivalents per gram of DW (mg Trolox/g DW).

### 3.5. LC-QTOF-MS/MS Analysis

The samples from the UW, UE, EW, and EE extracts were dissolved in 50% acetonitrile solution and passed through a 0.22 μm filter membrane to detect phenolic compounds. The compound analysis was performed on a LC-ESI-Q-TOF system (AB Sciex, Framingham, MA, USA) with slight modifications to the previously described method [42,43]. Chromatography separation was achieved on a Kinetex C18 column (100 × 4.6 mm, 2.6 μm) with a flow rate of 0.3 mL/min, an injection volume of 10 μL, and a column temperature of 35 °C in ESI^-^ mode. The mobile phase consisted of solution A (5 mM of ammonium acetate in water) and solution B (5 mM of ammonium acetate in 10% water/acetonitrile). Gradient elution was carried out as follows: 0–17 min, 5% solution B-50% solution B; 17–20 min, 50% solution B-95% solution B; 20–25 min, 95% solution B; 25–25.1 min, 95–5% solution B; 25.1–30 min, 5% solution B.

The mass spectrometric data were collected on a SCIEX X500R QTOF mass spectrometer (AB Sciex, Framingham, MA, USA) coupled with the SCIEX OS software 1.5 (AB, Milford, MA, USA) for data acquisition and processing. TOF MS and TOF MS/MS were scanned with a mass range of *m/z* 50–1000. Dynamic background subtraction (DBS) trigger information-dependent acquisition (IDA) was used to obtain MS/MS data. Furthermore, a maximum of 15 candidate ions was selected, and the intensity threshold exceeded 100 cps. The electrospray ion source temperature and spray voltage were set to 550 °C and −4500 V for the ESI^-^ modes, respectively. The declustering potential (DP), collision energy (CE), collision energy spread (CES), gas 1, gas 2, and curtain gas were 80 V, 35 V, ±15 V, 55 psi, 60 psi, and 35 psi, respectively. The accurate mass and composition for fragment ions were obtained. Compounds structures were then analyzed using the MassBank database (https://massbank.eu/MassBank/ (accessed on 20 June 2021) and databases built on standard compounds.

### 3.6. Statistical Analysis

SPSS v21.0 (IBM Corporation, New York, NY, USA) was used for statistical analyses. By using the ANOVA test and Duncan test, the results were analyzed for the difference between the variance and the means. In all tests, the differences between results were regarded as significant at *p* < 0.05.

## 4. Conclusions

In conclusion, the UE daylily extract had a higher total phenol content and stronger antioxidant activity than the EE, EW, or UW extracts. Thus, the combination of ultrasonic assistance and 50% ethanol solvent was effective for the extraction of phenolic compounds from daylily. Furthermore, 26 phenolic compounds including eight phenolic acids and 18 flavonoids were identified in the four extracts using LC-QTOF-MS/MS. According to multivariate statistical analysis, six differential phenolic compounds (chlorogenic acid, p-coumaric acid, naringenin, avicularin, kaempferol and isorhamnetin) were analyzed and quantified. Moreover, the UE extract exhibited the highest contents of the six differential compounds among the four extracts. 

## Figures and Tables

**Table 1:** Types of phenolic compounds identified using LC-QTOF-MS/MS.

No.	RT (min)	Identification	Formula	[M − H]^−^ (*m/z*)	MS^2^ (*m/z*)	Occurrence			
						UW	UE	EW	EE
1	3.51	Vanillin	C_8_H_8_O_3_	151.0401	136.0164, 92.0260		√		√
2	4.71	Robinin	C_33_H_40_O_19_	739.0256	285.0312	√	√	√	√
3	6.68	Kaempferol-3-O-rutinoside	C_27_H_30_O_15_	593.1478	327.0507, 285.0379, 255.0287	√	√	√	√
4	6.74	Isorhamnetin-3-O-neohesperpsode	C_28_H_32_O_16_	623.1583	315.0488, 299.0185	√	√	√	√
5	8.17	Astragalin	C_21_H_20_O_11_	447.0912	284.0308, 255.0284, 227.0337		√	√	√
6	8.24	Isorhamnetin-3-glucoside	C_22_H_22_O_12_	477.1018	314.0416, 271.0238	√	√	√	√
7	8.62	Hesperetin	C_16_H_14_O_6_	301.0711	286.0486, 164.0114, 151.0034, 134.0371, 108.0216		√		√
8	8.66	Rutin	C_27_H_30_O_16_	609.1444	300.0266, 255.0298	√	√	√	√
9	8.68	Dihydrokaempferol	C_15_H_12_O_6_	287.0556	259.0604, 177.0553, 125.0234, 57.0341	√	√	√	√
10	8.74	Kaempferol-3-O-arabinoside	C_20_H_18_O_10_	417.0807	284.0308, 255.0286, 227.0337	√	√	√	√
11	8.83	Quercitrin	C_21_H_20_O_11_	477.0926	314.0425, 271.0243		√		√
12	8.92	Naringenin	C_15_H_12_O_5_	271.0605	151.0031, 119.0494, 107.0132, 66.0025		√	√	√
13	10.14	Hyperoside	C_21_H_20_O_12_	463.089	300.0264, 255.0293	√	√	√	√
14	10.65	Avicularin	C_20_H_18_O_11_	433.0776	300.0263, 271.0249	√	√	√	√
15	11.26	Salicylic acid	C_7_H_6_O_3_	137.0231	93.0342, 65.0389	√	√	√	√
16	11.53	Isorhamnetin	C_16_H_12_O_7_	315.0541	300.0266, 151.0025		√		
17	11.63	Kaempferol	C_15_H_10_O_6_	285.041	255.0884		√		
18	14.5	Quercetin	C_15_H_10_O_7_	301.034	158.0423, 138.0283	√	√	√	√
19	14.85	Artemisinin	C_16_H_12_O_6_	299.2023	183.0123	√		√	
20	15.99	P-Coumaroylquinic acid	C_16_H_18_O_8_	191.054	173.0438, 163.0380, 119.0485	√	√	√	√
21	17.45	4-Hydroxybenzoic acid	C_7_H_6_O_3_	137.0228	93.0335, 65.0386	√	√	√	√
22	17.52	Vanillic acid	C_8_H_8_O_4_	167.035	152.0091, 108.0203, 91.0199	√	√	√	√
23	19.28	P-coumaric acid	C_9_H_8_O_3_	104.0406	119.0493, 93.0338	√	√	√	√
24	26.53	Caffeic acid	C_9_H_8_O_4_	179.0358	135.0440, 91.0549		√		√
25	26.59	3,4-Dihydroxybenzoic acid	C_7_H_6_O_4_	153.0168	108.0205, 91.0186		√		√
26	27.37	Chlorogenic acid	C_16_H_18_O_9_	353.0819	191.0555, 179.0339, 135.0445, 111.0082		√	√	√

**Table 2:** Contents of six differential compounds (mg/100 g DW) of the four daylily extracts.

Differential Compounds	Content (mg/100 g DW)			
	UW	UE	EW	EE
Chlorogenic acid	nd	6.713 ± 0.097 ^a^	6.17 ± 0.153 ^c^	6.406 ± 0.174 ^b^
P-coumaric acid	0.950 ± 0 ^b^	1.091 ± 0.012 ^a^	0.950 ± 0 ^b^	0.952 ± 0 ^b^
Kaempferol	nd	9.592 ± 0.167	nd	nd
Avicularin	nd	0.598 ± 0.001 ^a^	0.589 ± 0 ^c^	0.592 ± 0 ^b^
Naringenin	nd	2.759 ± 0.075 ^a^	2.059 ± 0.020 ^c^	2.456 ± 0.034 ^b^
Isorhamnetin	nd	2.126 ± 0.015	nd	nd

All test data are expressed as mean ± standard deviation and different letters represent significant differences (*p* < 0.05). The stats letters are compared between samples in rows. nd = not detected.

## Data Availability

The authors declare that all the data supporting the findings of this study are available within the article.

## Supplementary Materials

The following supporting information can be downloaded at: https://www.mdpi.com/article/10.3390/molecules27092964/s1, Table S1: The yield of the four extracts.; Figure S1: MS/MS spectra of daylily (*Hemerocallis citrina* Baroni) using LC-QTOF-MS/MS.

## Sample Availability

Samples of the compounds are not available from the authors.
