# Supplementary material for: Exploring the Extraction Methods of Phenolic Compounds in Daylily (Hemerocallis citrina Baroni) and Its Antioxidant Activity
Source: Molecules. 2022 May 5;27(9):2964. doi: 10.3390/molecules27092964 (PMC9101449; doi:10.3390/molecules27092964)
Supplement: Supplementary file 1 [file molecules-27-02964-s001.zip › molecules-1653423-supplementary.pdf]

molecules

MDPI

*Article*

# Exploring the Extraction Methods of Phenolic Compounds in Daylily (*Hemerocallis citrin*a Baroni) and Its Antioxidant Activity

**Zhilin Hao, Li Liang, He Liu, Yi Yan * and Yuyu Zhang**

Beijing Key Laboratory of Flavor Chemistry, School of Light Industry, Beijing Technology and Business University (BTBU),
Beijing 100048, China; hzl15716324037@163.com (Z.H.); gcfll@126.com (L.L.); liuhe6660@163.com (H.L.); zhangyuyu@btbu.edu.cn (Y.Z.)
* Correspondence: yanyi@btbu.edu.cn; Tel.: +86-132-0630-0168

**Table S1.** The yield of the four extracts.

| Extraction method | UW | UE | EW | EE |
|---|---|---|---|---|
| Extraction yield (%) | 14.33±2.71[c] | 27.06±1.63[b] | 41.50±1.88[a] | 44.96±2.4[a] |

Different letters represent significant differences according to Duncan's test ($p < 0.05$). UW = ultrasound-assisted water extraction, UE = ultrasound-assisted ethanol extraction, EW = enzymatic-assisted water extraction, EE = enzymatic-assisted ethanol extraction.

3.51 Vanillin $C_8H_8O_3$

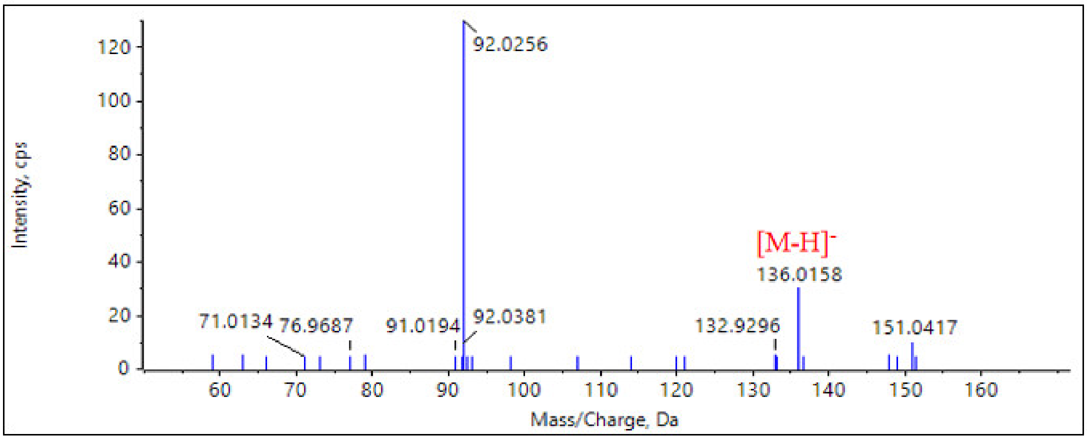


**4.7**1 Robinin $C_{33}H_{40}O_{19}$.

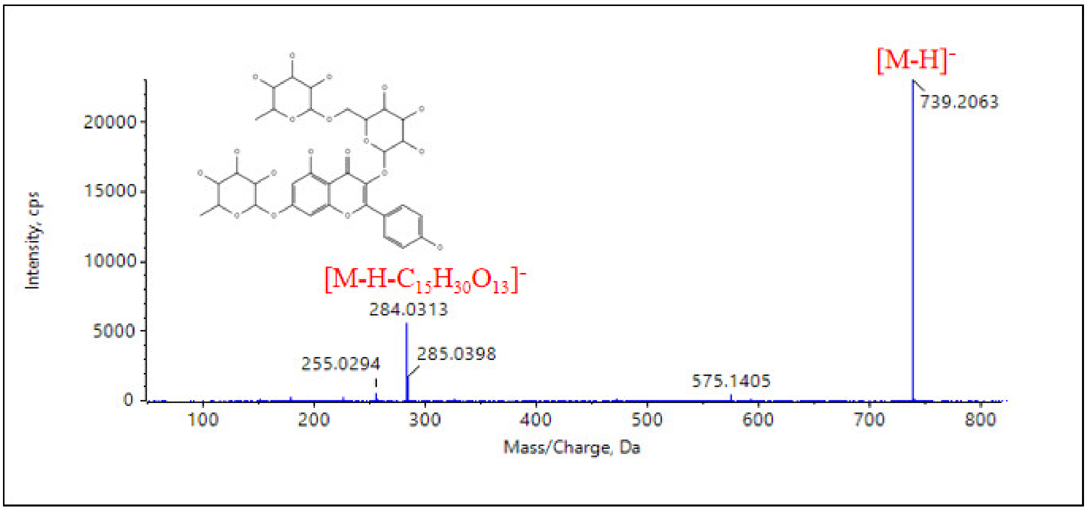


6.68 Kaempferol-3-O-rutinoside $C_{27}H_{30}O_{15}$

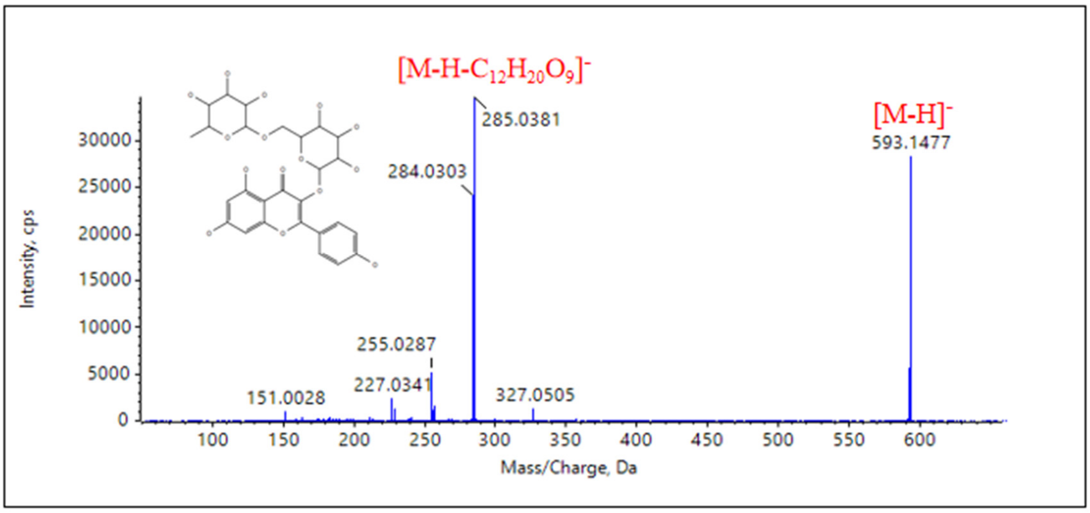


6.74 Isorhamnetin-3-O-neohesperpsode $C_{28}H_{32}O_{16}$

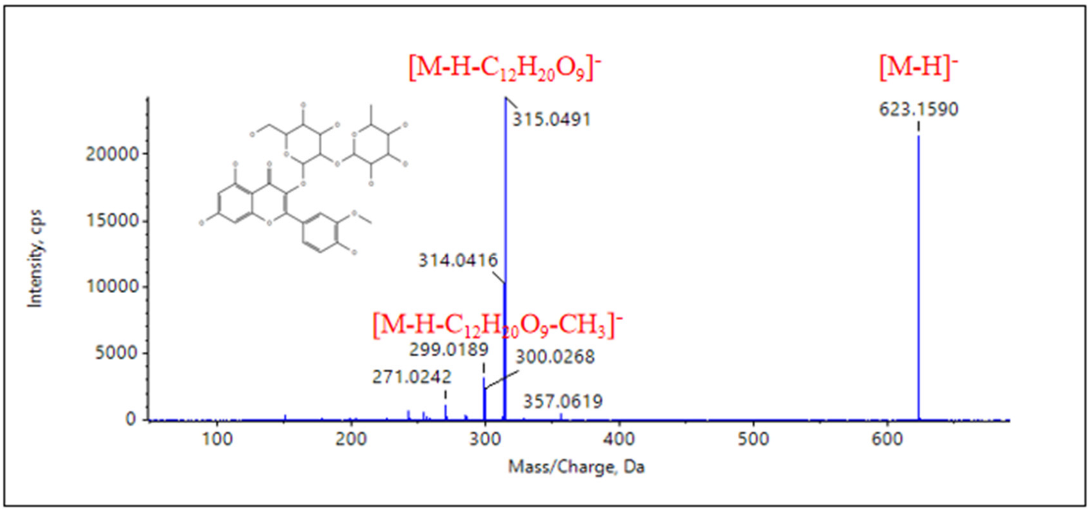


8.17 Astragalin $C_{21}H_{20}O_{11}$

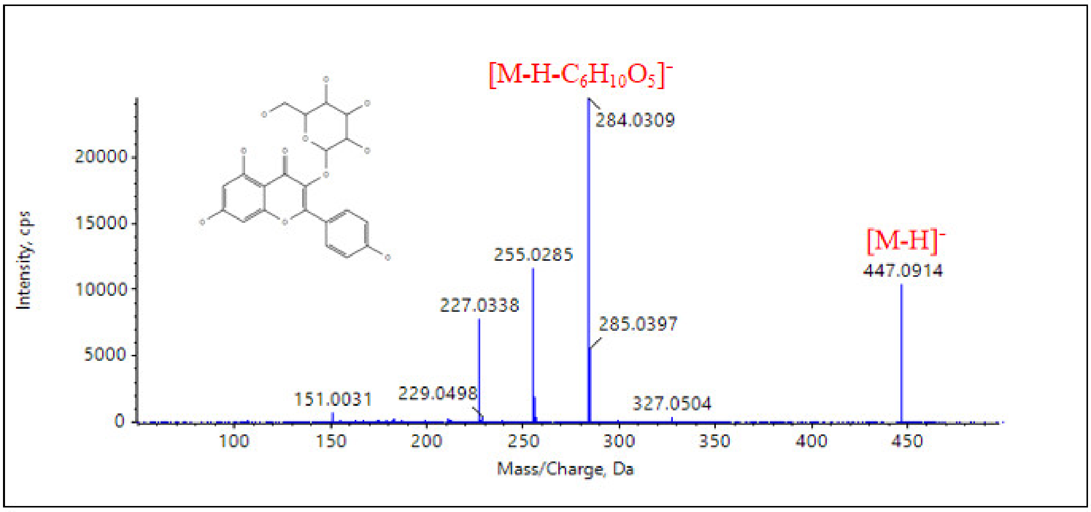


8.24 Isorhamnetin-3-glucoside $C_{22}H_{22}O_{12}$

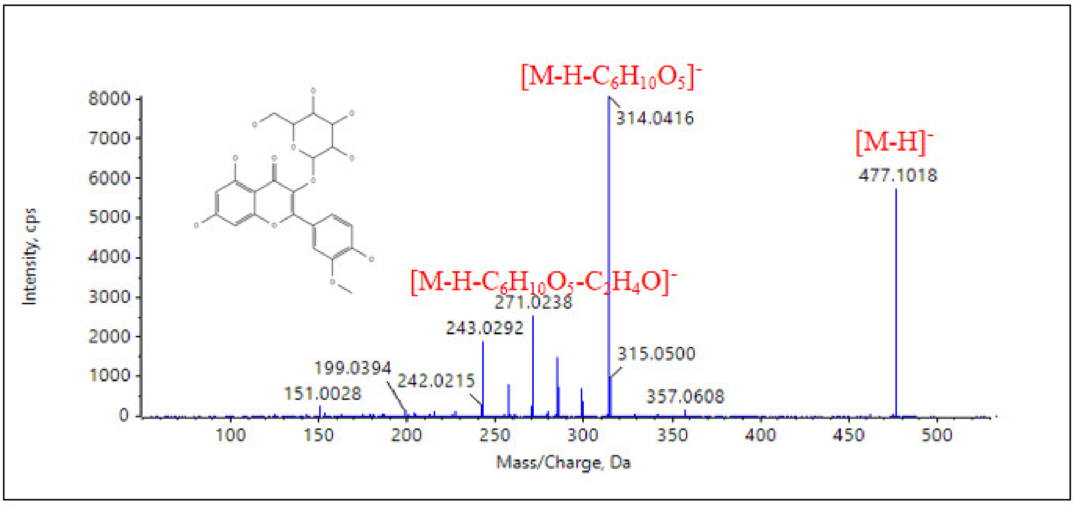

8.62 Hesperetin $C_{16}H_{14}O_6$

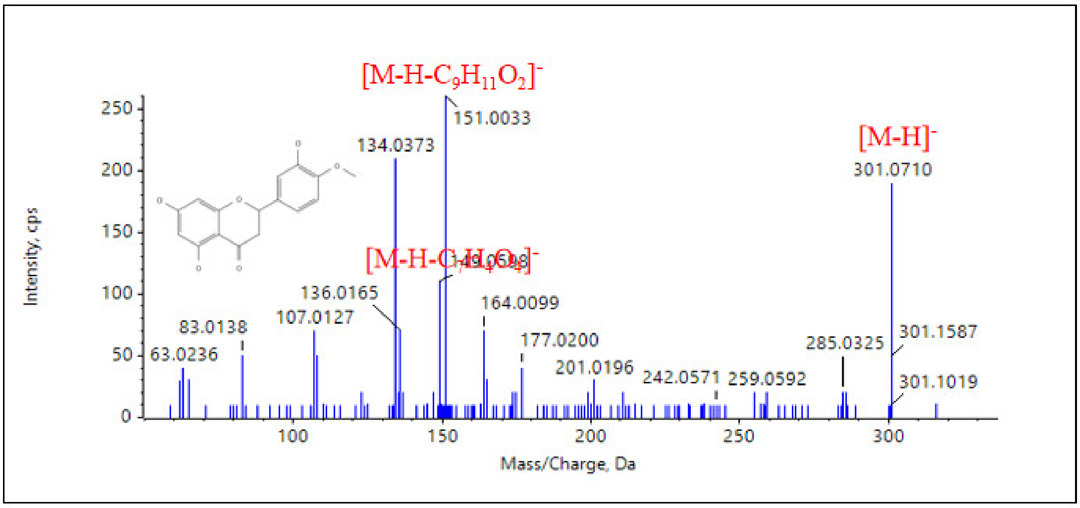


8.66 Rutin $C_{27}H_{30}O_{16}$

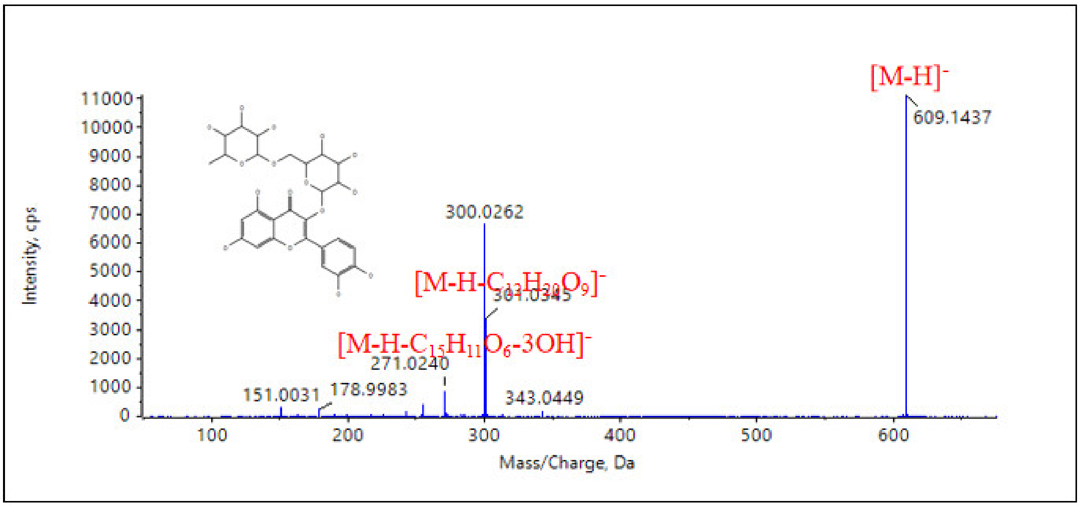


8.68 Dihydrokaempferol $C_{15}H_{12}O_6$

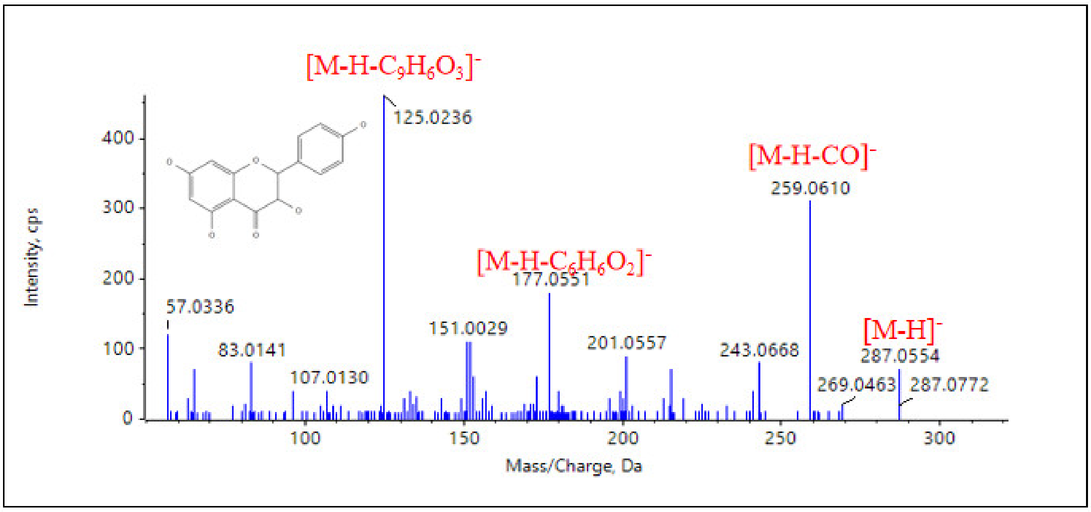

8.74 Kaempferol-3-O-arabinoside $C_{20}H_{18}O_{10}$

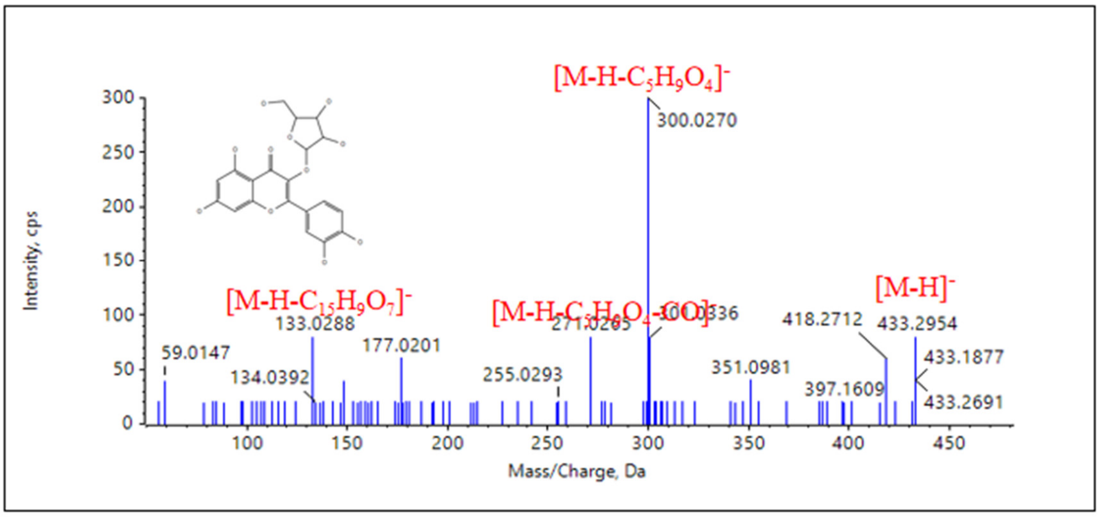


8.83 Quercitrin $C_{21}H_{20}O_{11}$

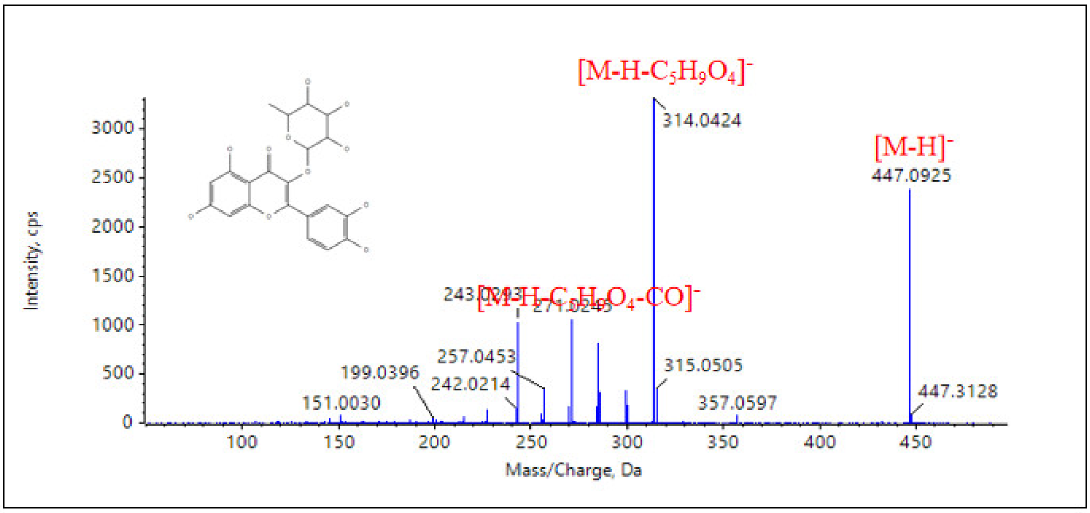


8.92 Naringenin $C_{15}H_{12}O_5$

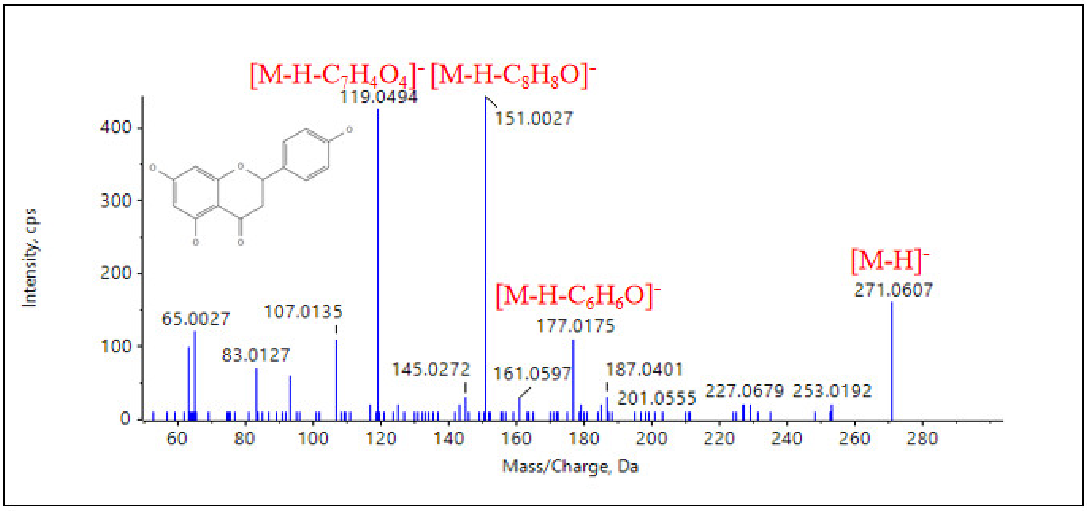

10.14 Hyperoside $C_{21}H_{20}O_{12}$

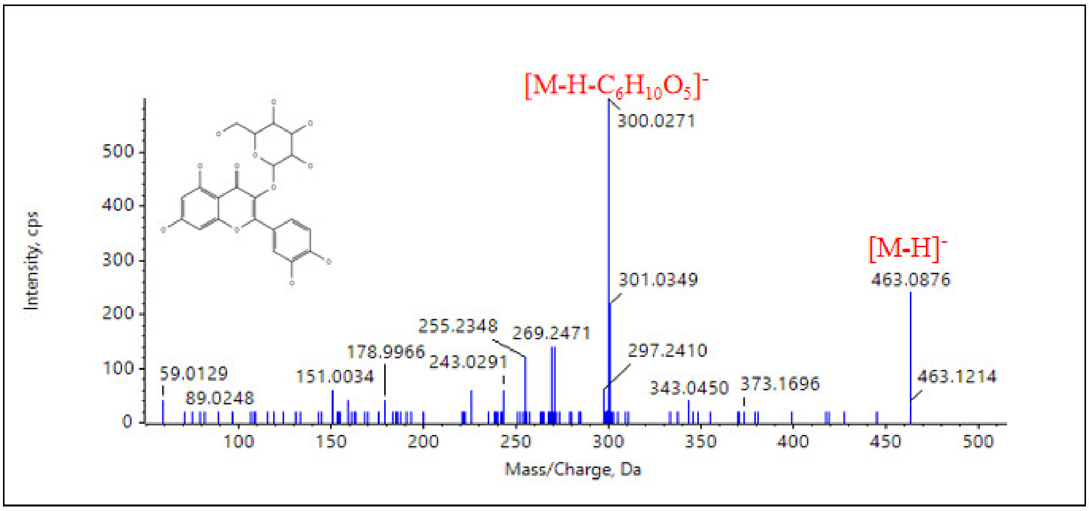


10.65 Avicularin $C_{20}H_{18}O_{11}$

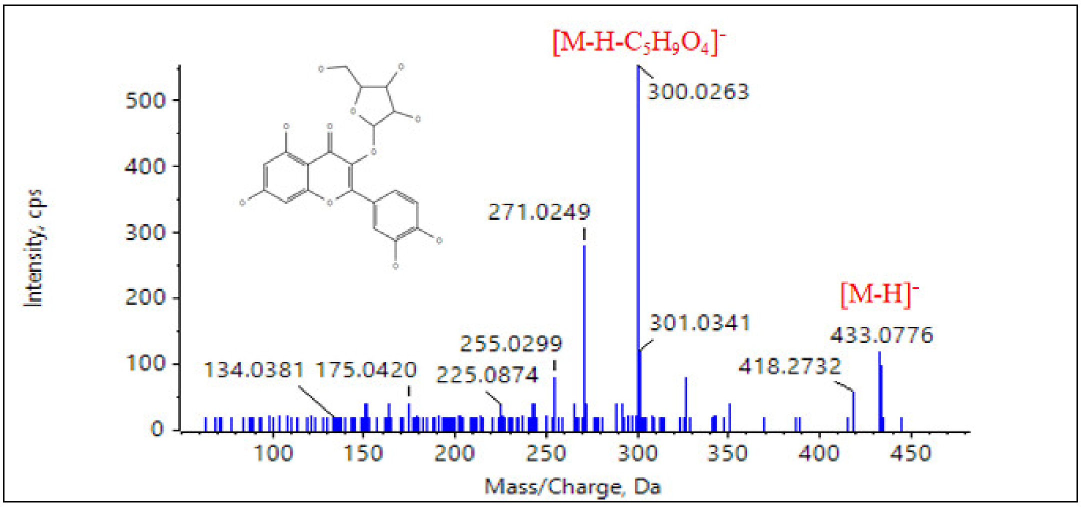


11.26 Salicylic acid $C_7H_6O_3$

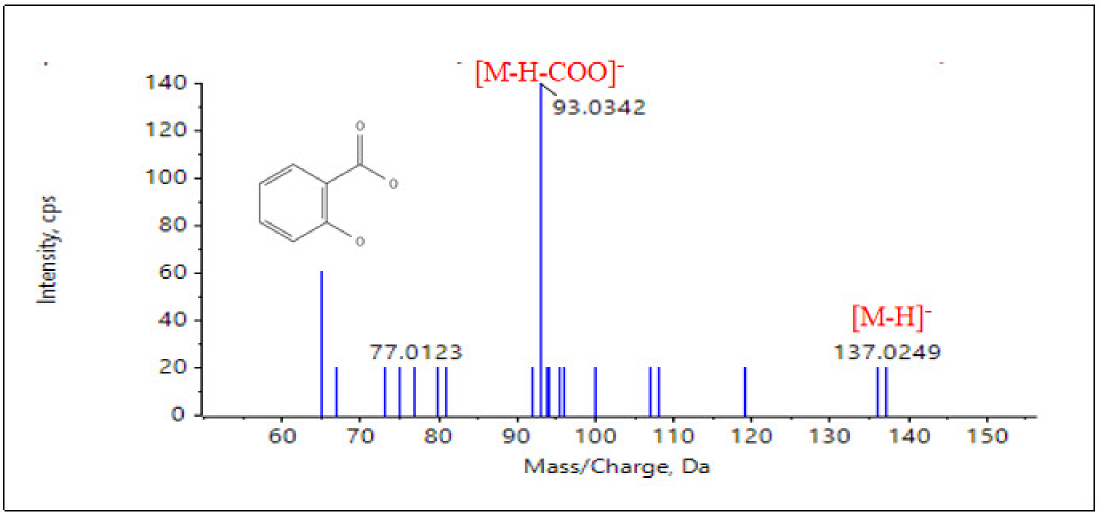

11.53 Isorhamnetin $C_{16}H_{12}O_7$

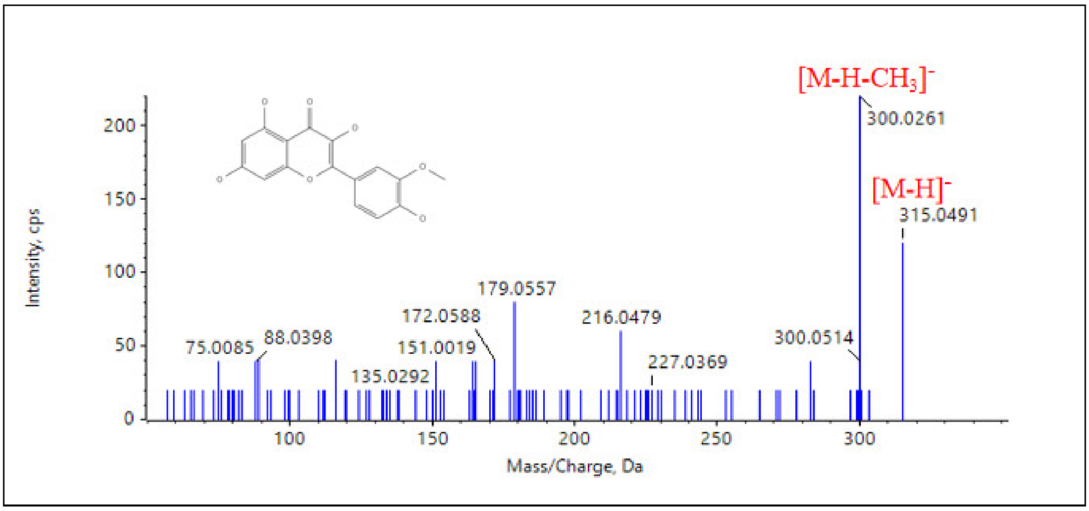


11.63 Kaempferol $C_{15}H_{10}O_6$

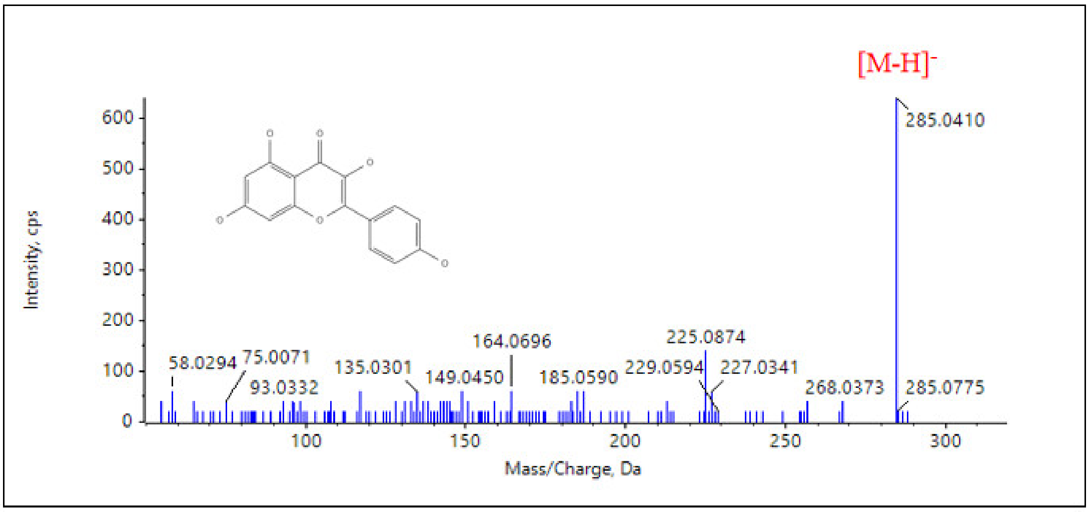


14.5 Quercetin $C_{15}H_{10}O_7$

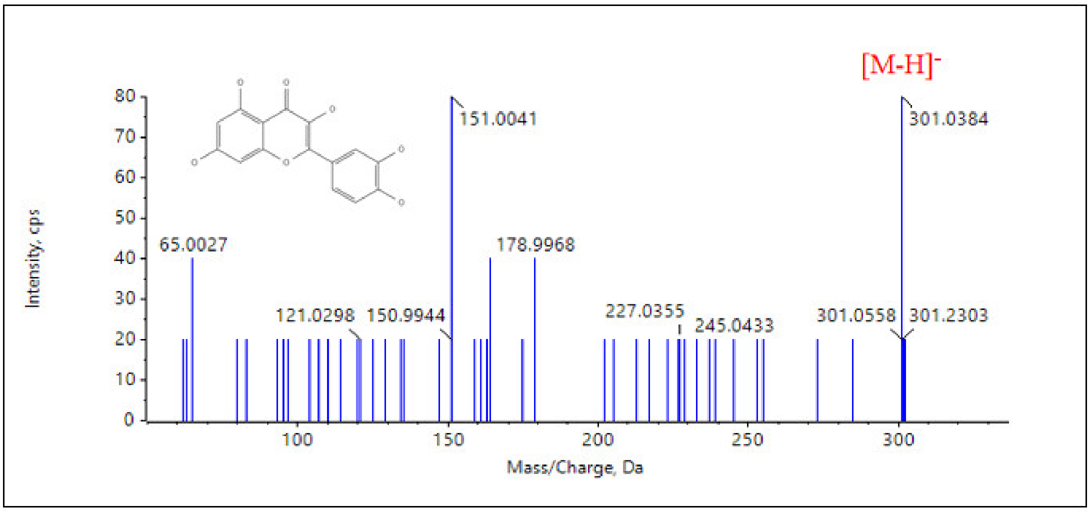

14.85 Artemisinin $C_{16}H_{12}O_6$

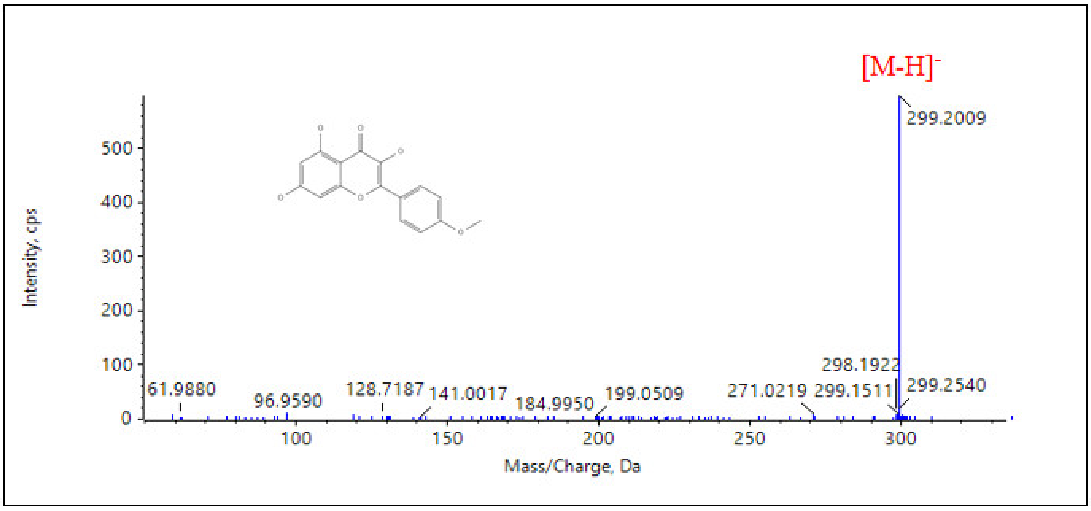


15.99 P-Coumaroylquinic acid $C_{16}H_{18}O_8$

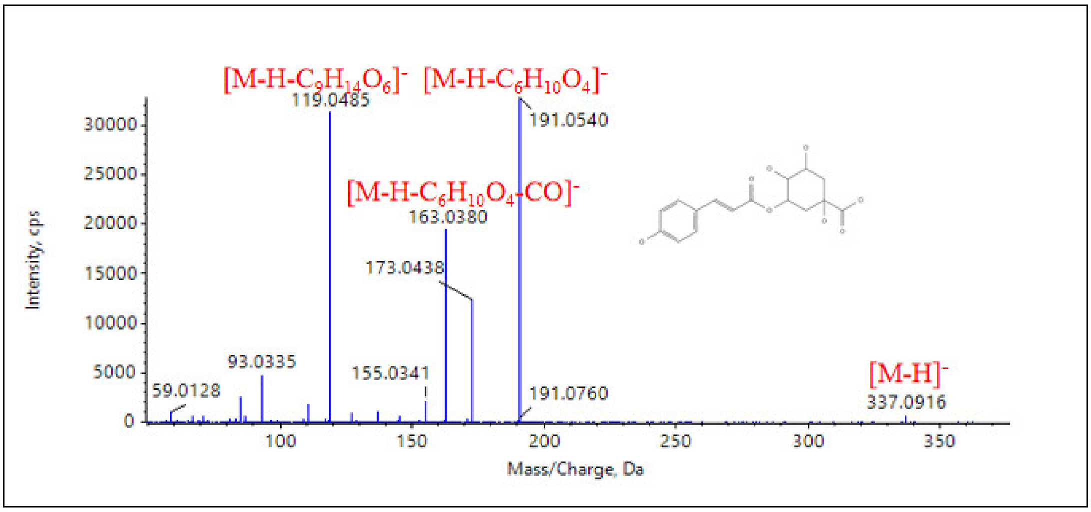


17.45 4-Hydroxybenzoic acid $C_7H_6O_3$

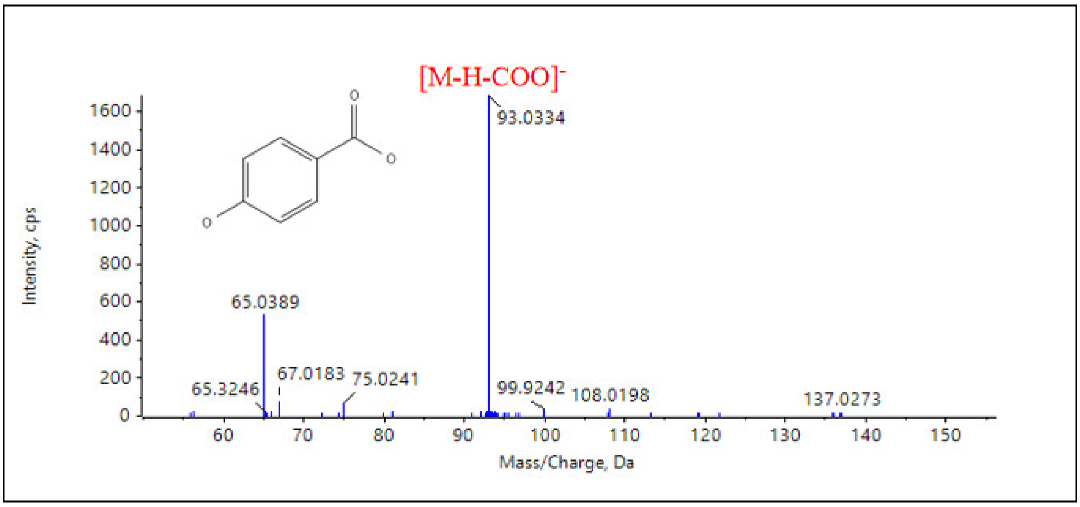

17.52 Vanillic acid $C_8H_8O_4$

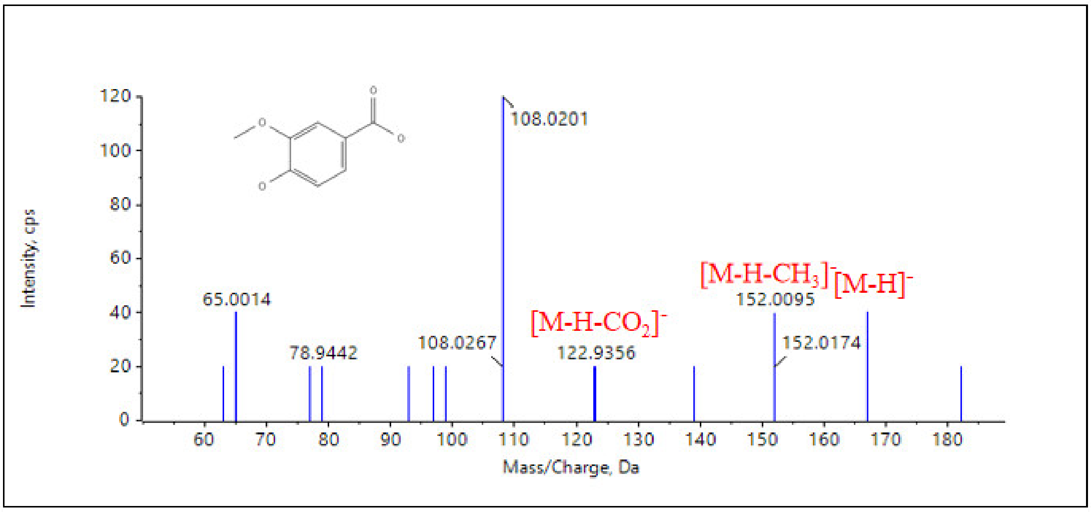


19.28 P-coumaric acid $C_9H_8O_3$

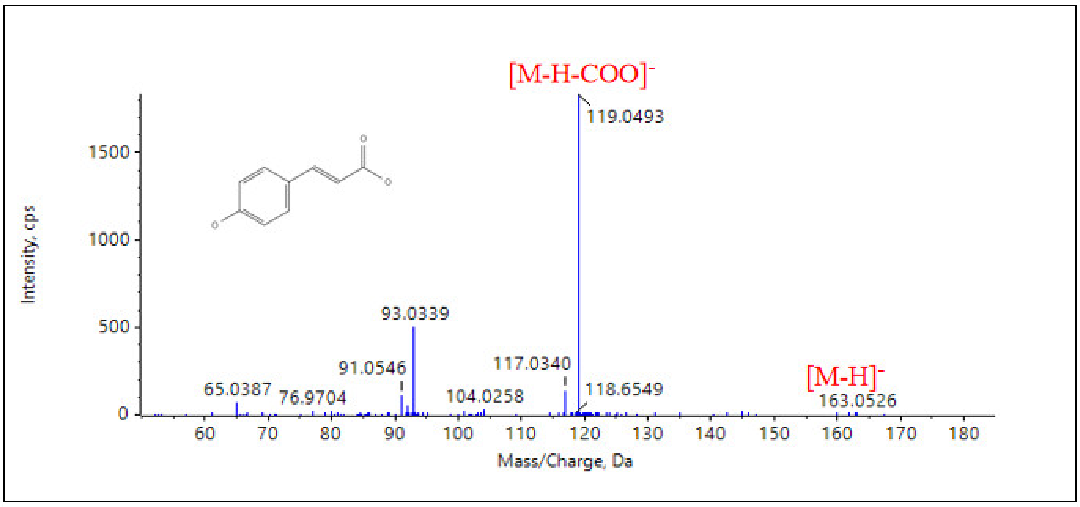


26.53 Caffeic acid $C_9H_8O_4$

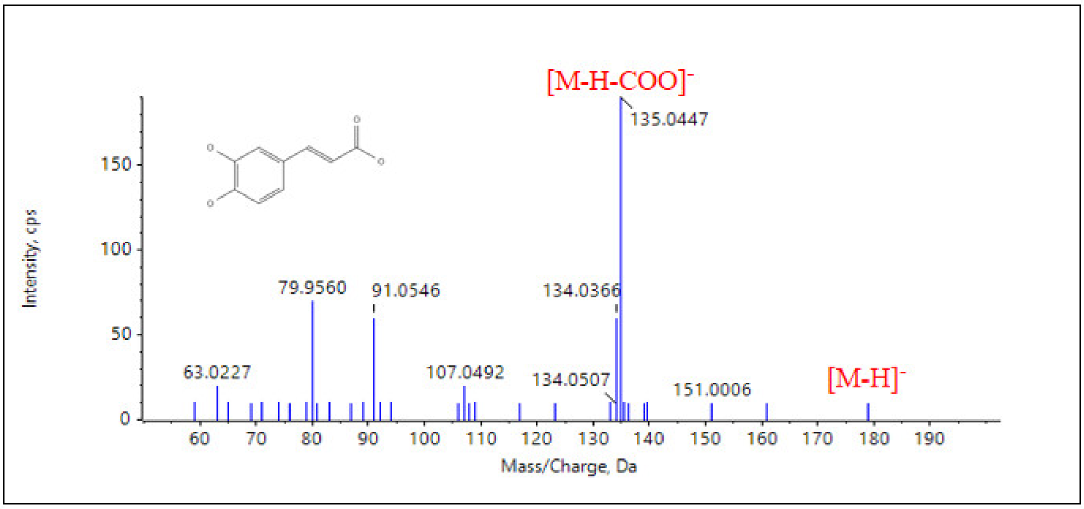

26.59 3,4-Dihydroxybenzoic acid $C_7H_6O_4$

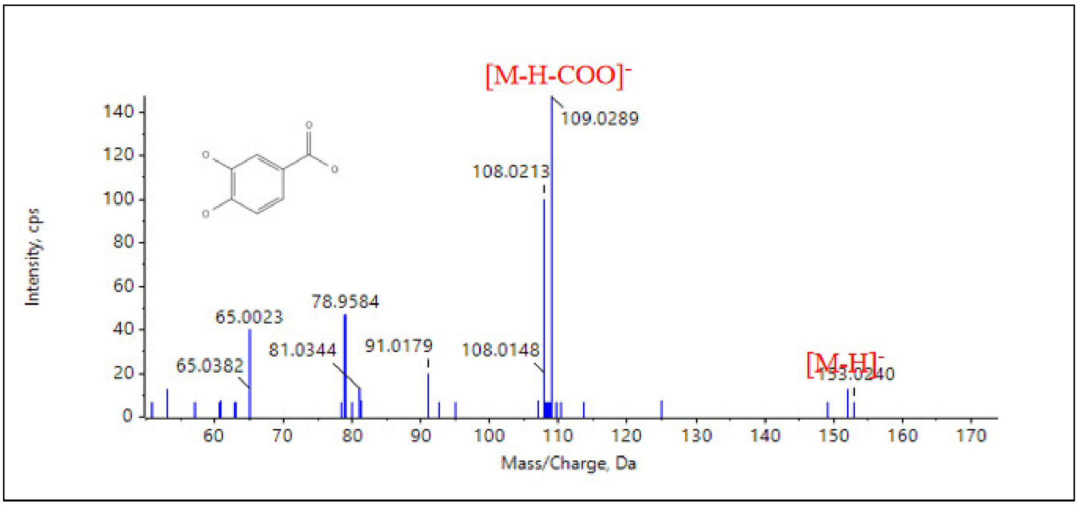


27.37 Chlorogenic acid $C_{16}H_{18}O_9$

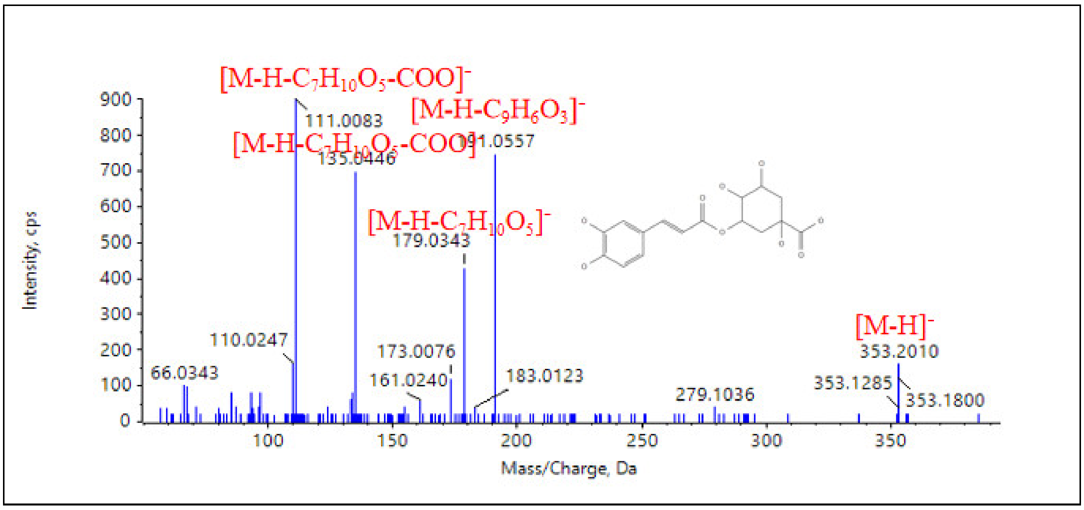


**Figure S1.** MS/MS spectra of daylily (*Hemerocallis citrin*a Baroni) using LC-QTOF-MS/MS.
